# Supplementary material for: Cancer of Unknown Primary (CUP): genetic evidence for a novel nosological entity? A case report
Source: EMBO Mol Med. 2020 Jun 8;12(7):e11756. doi: 10.15252/emmm.201911756 (PMC7338804; doi:10.15252/emmm.201911756)
Supplement: Supplementary file 1 — Expanded View Figures PDF [file EMMM-12-e11756-s001.pdf]

Expanded View Figures

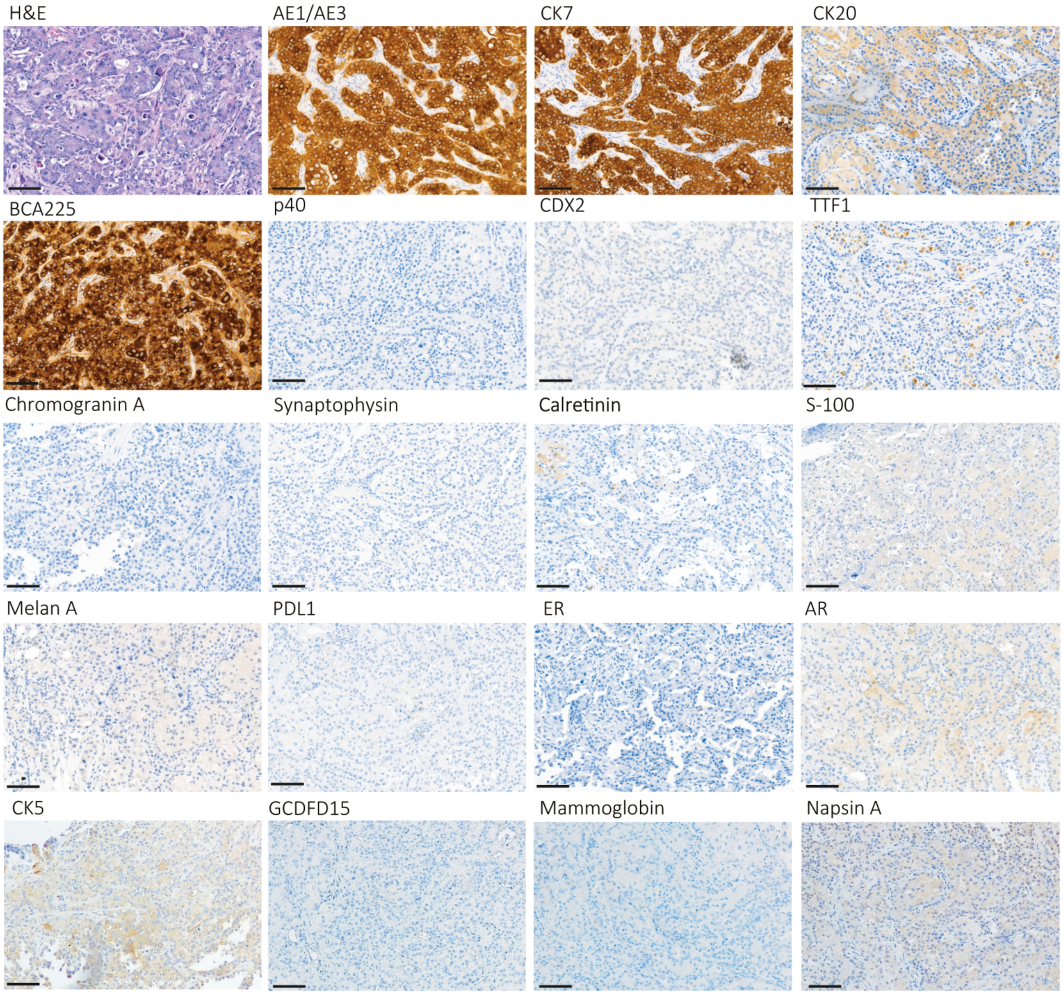

Figure EV1. IHC analysis.

Nineteen IHC reactions performed on the breast core biopsy performed during the *ad excludendum* CUP diagnosis. H&E: hematoxylin and eosin. Scale bar: 50 µm.

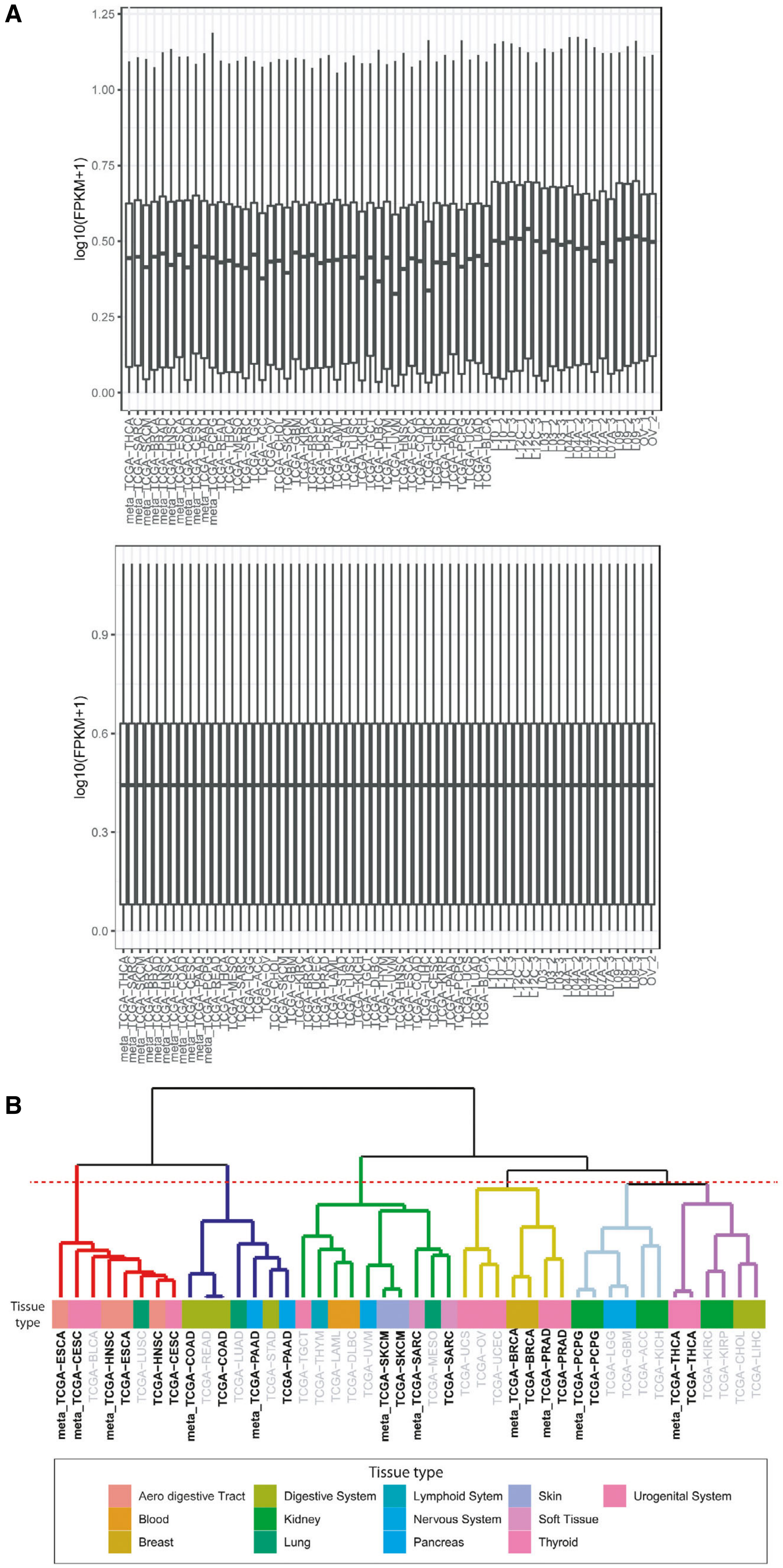

Figure EV2. Quantile normalization and hierarchical clustering.

A TCGA transcriptional profiles were normalized using the normalize quantiles function of preprocessCore package in the R statistical environment v3.6. Each box represents the upper and lower quartiles, while the central short black line within each box represents the median; whiskers indicate variability outside the upper and lower quartiles.

B The hierarchical clustering analysis of TCGA primary tumors and metastases from known primaries (performed using median expression profiles) shows that each metastasis clusters close to its tumor of origin. In grey primary tumors for which metastases profiles were not available.

 © 2020 The Authors

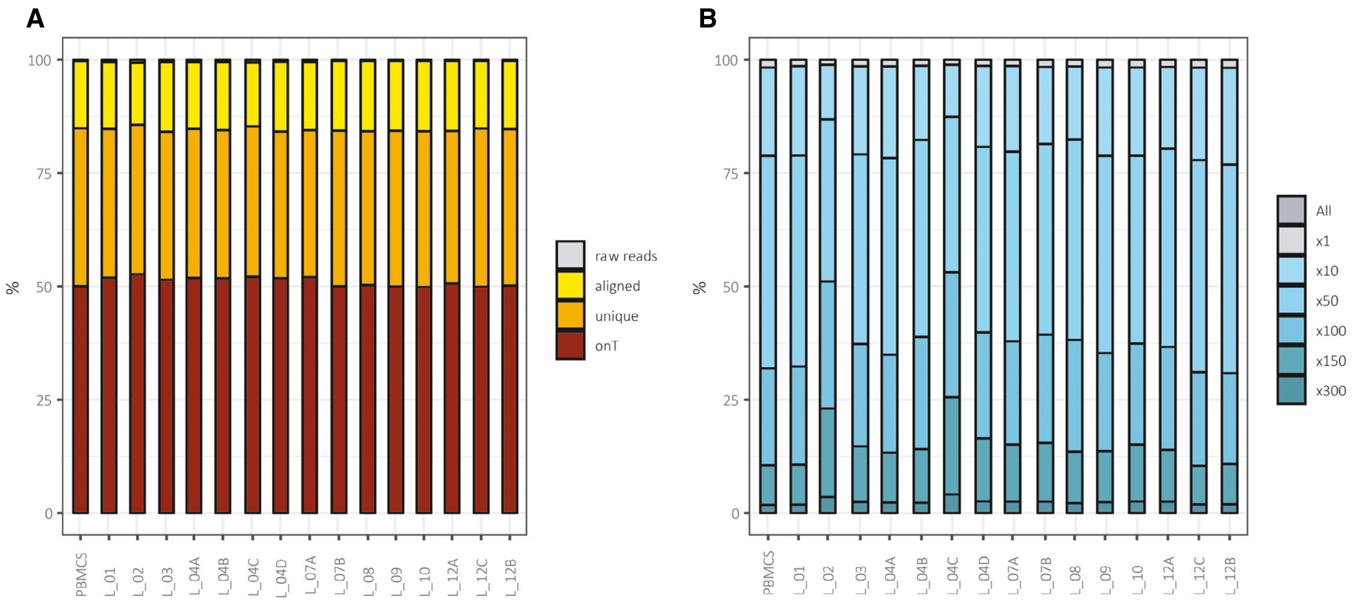

Figure EV3. Coverage details.

A Percentage of reads retained at each filtering steps over the total number of aligned sequencing reads. On the *x*-axis are aligned samples. onT, on target.
B Percentage of targeted base pairs that were sequenced at different depth of coverage. On the *x*-axis are aligned samples.

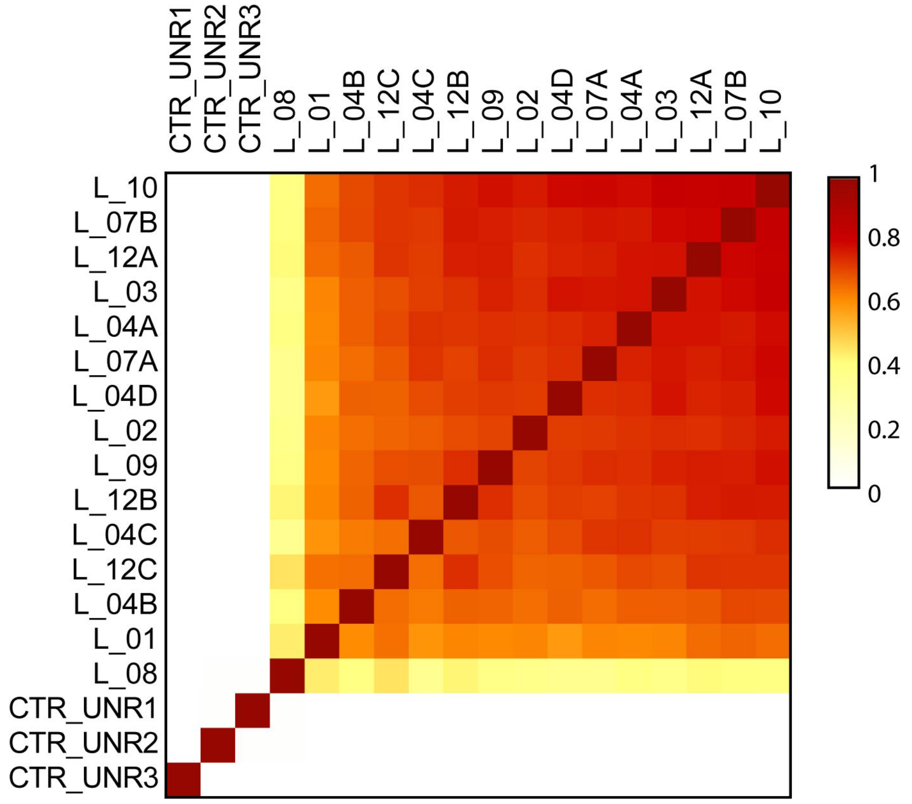

Figure EV4. Genetic similarity among metastases is specific.

The genetic similarity among fifteen CUP metastases was calculated using Jaccard index, as shown in Fig 3B. Here, data from WES of three unrelated metastases (CTR_UNR1, CTR_UNR2, and CRT_UNR3) were included. The genetic similarity among those controls and metastases of the CUP patient (measured as function of Jaccard index) was zero (white squares).

© 2020 The Authors

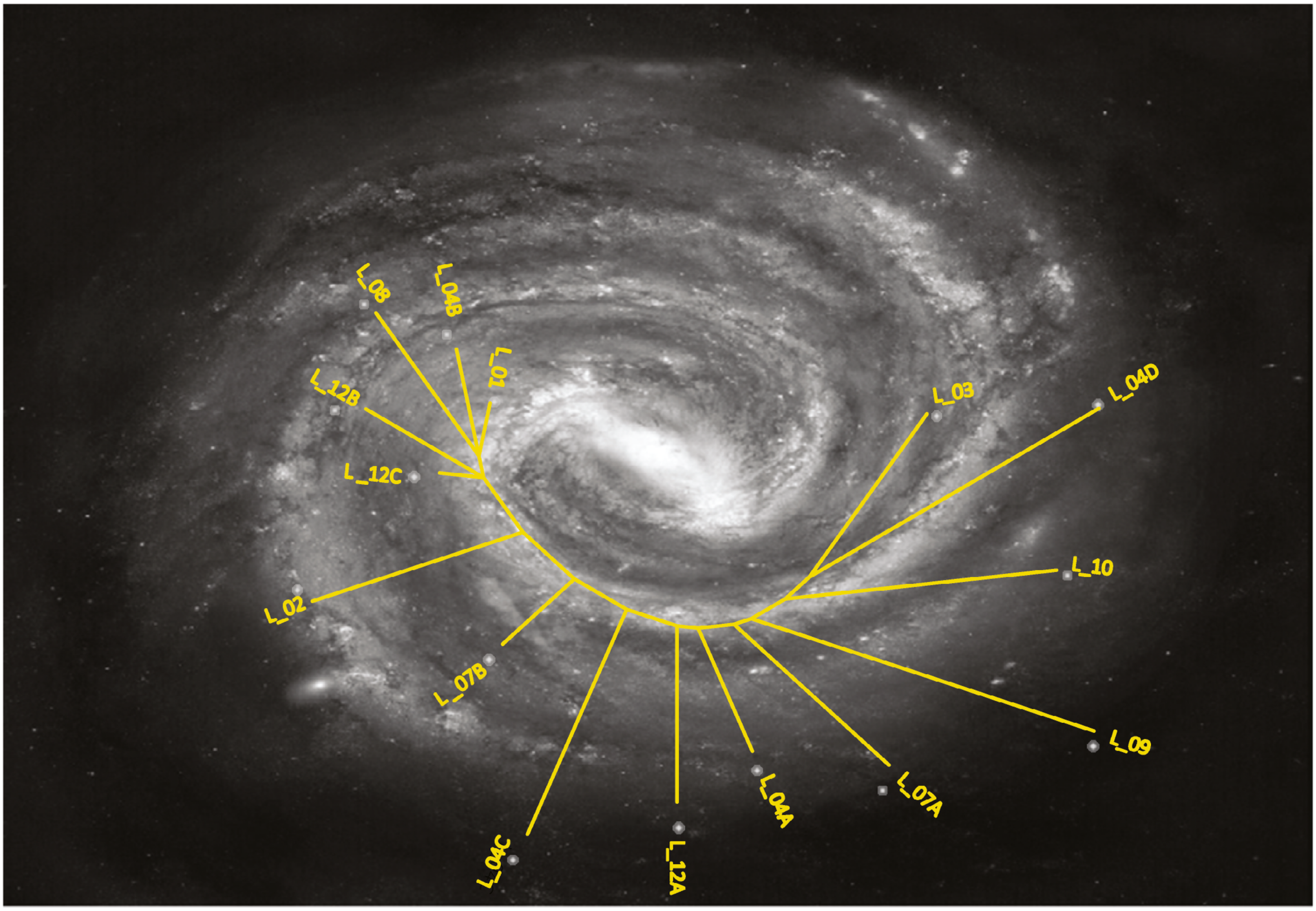

Figure EV5. Phylogenetic tree reconstruction.

The phylogenetic tree linking the fifteen metastases reconstructed by the Phylip tool (as described in Materials and Methods) is suggestive of an expansion pattern of a galaxy.

 © 2020 The Authors
