# Supplementary material for: Cancer of Unknown Primary (CUP): genetic evidence for a novel nosological entity? A case report
Source: EMBO Mol Med. 2020 Jun 8;12(7):e11756. doi: 10.15252/emmm.201911756 (PMC7338804; doi:10.15252/emmm.201911756)
Supplement: Supplementary file 2 — Table EV1 [file EMMM-12-e11756-s002.docx]

**Table EV1: Serum and immunohistochemistry (IHC) markers.**

| *Serum markers* |
| --- |
| Increased: carcinoembryonic antigen (CEA, ±). |
| Normal: alpha-fetoprotein (AFP), β2-microglobulin, carbohydrate antigen 19-9 (CA19.9), human chorionic gonadotropin (hCG), prostate specific antigen (PSA). |
| *IHC markers* |
| Positive: BCA225, cytokeratin 7 (CK7), cytokeratin 20 (CK20), cytokeratin AE1/AE3 (CK AE1/AE3). |
| Negative: androgen receptor (AR), caudal type homeobox 2 (CDX2), calretinin, chromogranin A, cytokeratin 5 (CK5), estrogen receptor (ER), gross cystic disease fluid protein 15 (GCDFP15), mammoglobin, melan A, napsin A, p40, programmed death ligand 1 (PDL1), synaptophysin, thyroid transcript factor-1 (TTF-1), S-100. |
