# Supplementary material for: Cancer of Unknown Primary (CUP): genetic evidence for a novel nosological entity? A case report
Source: EMBO Mol Med. 2020 Jun 8;12(7):e11756. doi: 10.15252/emmm.201911756 (PMC7338804; doi:10.15252/emmm.201911756)
Supplement: Supplementary file 3 — Table EV2 [file EMMM-12-e11756-s003.docx]

**Table EV2: Hot spot genetic alterations (mutations, amplifications and translocations).**

| *Hot spot genetic alterations* | |
| --- | --- |
| Mutations | **Status** |
| ABL1: G250E, Q252H, Y253H, Y253F, E255K, E255V, D276G, F311L, T315I, F317L, M351T, E355G, F359V, H396R | wild type |
| AKT1:E17K, Q43X, V167A, E319G, L357P, P388T, V461L | wild type |
| AKT2:S302G, R371H | wild type |
| BRAF: G464R, G464V/E, G466R, F468C, G469A, G469E, G469R, G469S, G469V, D594V/G, F595L, G596R, L597Q, L597R, L597S, L597V, T599I, V600E, V600K, V600L, V600R, K601N, K601E | wild type |
| CDK4: R24C, R24H | wild type |
| EGFR: R108K, T263P, A289V, G598V, E709A/G/V, E709K/H, G719A, G719S/C, E746_A750del, E746_T751del, E746_T751>A, E746_T751>S, E746_T751>V, E746_S752>V, E746_S752>D, E746_S752>A, E746_S752>I, L747S, L747_E749del, L747_A750del, L747_A750>P, L747_T751del, L747_T751>P, L747_S752del, L747_P753>Q, L747_P753>S, A750P, T751A, T751P, T751I, S752F, S752_I759del, P753Q, M766_A767insAI, S768I, V769_D770insASV, V769_D770insCV, D770_N771>AGG/N771>GF, D770_N771insG, D770_N771insGF, D770_N771insGL, D770fs*61/D770_N771insAPW, N771T, P772_H773insV, H773N, H773_V774insNPH, H773_V774insPH, H773_V774insH, V774_C775insHV, T790M, L858R, L861Q ERBB2 L755P, A775_G776insYVMA, G776S/LC, G776VC/VC, S779_P780insVGS, P780_Y781insGSP FGFR1 S125L, P252T FGFR3 G370C, Y373C, A391E, K650Q/E, K650T/M FLT3 D835H/Y, I836del HRAS G12V/D, G13C/R/S, Q61H/H, Q61L/R/P, Q61K JAK2 V617F KIT D52N, Y503_F504insAY, K550_K558del, P551_V555del, M552L, Y553_Q556del, W557R/R/G, K558_V560del, K558_E562del, V559del, V559D/A/G, V559I, V559_V560del, V560D/G, V560del, E561K, Y568D, Y570_L576del, L576P, D579del, F584S, P585P, K642E, D816H/Y, D816V, V825A, E839K KRAS G12A, G12C, G12D, G12F, G12R, G12S, G12V, G13V/D, A59T, Q61E/K, Q61H/H, Q61L/R/P MET R970C, T992I, Y1230C, Y1235D, M1250T NRAS G12C/R/S, G12V/A/D, G13C/R/S, G13V/A/D, A18T, Q61E/K, Q61H, Q61L/R/P PDGFRA V561D, S566_E571>K, T674I, F808L, D842_H845del, D842V, I843_D846del, I843_S847>T, D846Y, N870S, D1071N | wild type |
| ERBB2: L755P, A775_G776insYVMA, G776S/LC, G776VC/VC, S779_P780insVGS, P780_Y781insGSP | wild type |
| FGFR1: S125L, P252T | wild type |
| FGFR3: G370C, Y373C, A391E, K650Q/E, K650T/M | wild type |
| FLT3: D835H/Y, I836del | wild type |
| HRAS: G12V/D, G13C/R/S, Q61H/H, Q61L/R/P, Q61K | wild type |
| JAK2: V617F | wild type |
| KIT: D52N, Y503_F504insAY, K550_K558del, P551_V555del, M552L, Y553_Q556del, W557R/R/G, K558_V560del, K558_E562del, V559del, V559D/A/G, V559I, V559_V560del, V560D/G, V560del, E561K, Y568D, Y570_L576del, L576P, D579del, F584S, P585P, K642E, D816H/Y, D816V, V825A, E839K | wild type |
| KRAS: G12A, G12C, G12D, G12F, G12R, G12S, G12V, G13V/D, A59T, Q61E/K, Q61H/H, Q61L/R/P | wild type |
| MET: R970C, T992I, Y1230C, Y1235D, M1250T | wild type |

| NRAS: G12C/R/S, G12V/A/D, G13C/R/S, G13V/A/D, A18T, Q61E/K, Q61H, Q61L/R/P | wild type | |
| --- | --- | --- |
| PDGFRA: V561D, S566_E571>K, T674I, F808L, D842_H845del, D842V, I843_D846del, I843_S847>T, D846Y, N870S, D1071N | wild type | |
| PIK3CA: R38H, R88Q, N345K, C420R, P539R, E542K, E545K, Q546K, H701P, C901F, M1043I, H1047R/L, H1047Y | wild type | |
| RET: E632_L633del, C634R, C634W, C634Y, A664D, M918T | wild type | |
| **Amplifications** | **Status** | |
| HER2 | | wild type |
| EGFR | | wild type |
| MET | | wild type |
| **Translocations** | **Status** | |
| ALK (2p23) | | wild type |
| ROS1 (6q22) | | wild type |

Mutations were scrutinised by OncoCarta^TM^; amplifications and translocations by FISH (fluorescence *in situ* hybridization).
