# Supplementary material for: Cancer of Unknown Primary (CUP): genetic evidence for a novel nosological entity? A case report
Source: EMBO Mol Med. 2020 Jun 8;12(7):e11756. doi: 10.15252/emmm.201911756 (PMC7338804; doi:10.15252/emmm.201911756)
Supplement: Supplementary file 4 — Table EV3 [file EMMM-12-e11756-s004.docx]

**Table EV3: Antibodies and probes used for the *ad excludendum* analysis.**

| *IHC* | | | |
| --- | --- | --- | --- |
| Antibody (clone) | **Source** | | **Dilution** |
| CK7 (OV-TL 12/3) | | Dako | 1:80 |
| CK5 (XM2G) | | Thermo-scientific | 1:150 |
| CK20 (Ks20.8) | | Dako | 1:60 |
| CK AE1/AE3 (AE1-AE3) | | Dako | 1:200 |
| TTF-1 (8G7G3/1) | | Diagnostic biosystems | 1:50 |
| P40 (BC28) | | Biocare | 1:100 |
| Napsin A (TMU-Ad02) | | ARP (American Research Product) | 1:120 |
| CDX2 (DAK-CDX2/Monosan; AMT28) | | Dako | rtu |
| GCDFP15 (23A3) | | Dako | 1:100 |
| Mammoglobin (304-1A5) | | Dako | 1:100 |
| BCA225 (CU18) | | Abcam | 1:50 |
| Chromogranin A (%H7) | | Leica | rtu |
| Synaptophysin (DAK-SYNAP) | | Dako | 1:2 |
| Calretinin (Polyclonal) | | Swant Inc. | 1:500 |
| Melan-A (A103) | | Leica | rtu |
| S100 (Polycolnal) | | Dako | 1:2000 |
| ER (EP1) | | Dako | 1:80 |
| AR (AR441) | | Biocare | 1:40 |
| PDL1 (22c3) | | Dako | rtu |
| *FISH* | | | |
| Antibody | | **Source** | **Dilution** |
| HER2 IQFISH pharmDX | | Dako | rtu |
| EGFR/CEP7 dual color probe | | Vysis-Abbott | rtu |
| MET/CEN7 dual color probe | | ZytoVision | rtu |
| ALK dual color break apart probe | | ZytoVision | rtu |
| ROS-1 dual color break apart probe | | ZytoVision | rtu |
| DAPI I | | Vysis | rtu |

AFP: alpha-fetoprotein; AR: androgen receptor; CA19.9: carbohydrate antigen 19-9; CDX2: caudal type homeobox 2; CEA: carcinoembryonic antigen; CK: cytokeratin; ER: estrogen receptor; GCDFP15: gross cystic disease fluid protein 15; hCG: human chorionic gonadotropin; IHC: immunohistochemistry; PDL1: programmed death ligand 1; PSA: prostate specific antigen; TTF-1: thyroid transcript factor-1; rtu: ready-to-use.
